# Characteristics and outcomes of patients with eclampsia and severe pre-eclampsia in a rural hospital in Western Tanzania: a retrospective medical record study

**DOI:** 10.1186/s12884-015-0649-2

**Published:** 2015-09-08

**Authors:** Rob Mooij, Joseph Lugumila, Masumbuko Y. Mwashambwa, Ipyana H. Mwampagatwa, Jeroen van Dillen, Jelle Stekelenburg

**Affiliations:** Ndala Hospital, 15 Ndala, Tabora, Tanzania; Department of Obstetrics and Gynaecology, Maastricht University Medical Centre, P. Debyelaan 25, 6229 HX Maastricht, The Netherlands; College of Health Sciences, University of Dodoma, 395 Dodoma, Tanzania; Department of Obstetrics and Gynaecology, Radboud University Medical Centre, Geert Grooteplein-Zuid 10, 6525 GA Nijmegen, The Netherlands; Department of Obstetrics and Gynaecology, Leeuwarden Medical Centre, Henri Dunantweg 2, 8934 AD Leeuwarden, The Netherlands

## Abstract

**Background:**

Eclampsia and pre-eclampsia are well-recognized causes of maternal and neonatal mortality in low income countries, but are never studied in a district hospital. In order to get reliable data to facilitate the hospital’s obstetric audit a retrospective medical record study was performed in Ndala Hospital, Tanzania.

**Methods:**

All patients diagnosed with severe pre-eclampsia or eclampsia between July 2011 and December 2012 were included. Medical records were searched immediately following discharge or death. General patient characteristics, medical history, obstetrical history, possible risk factors, information about the current pregnancy, antenatal clinic attendance and prescribed therapy before admission were recorded. Symptoms and complications were noted. Statistical analysis was done with Epi Info®.

**Results:**

Of the 3398 women who gave birth in the hospital 26 cases of severe pre-eclampsia and 55 cases of eclampsia were diagnosed (0.8 and 1.6 %). Six women with eclampsia died (case fatality rate 11 %). Convulsions in patients with eclampsia were classified as antepartum (44 %), intrapartum (42 %) and postpartum (15 %). Magnesium was given in 100 % of patients with eclampsia and was effective in controlling convulsions. Intravenous antihypertensive treatment was only started in 5 % of patients. Induction of labour was done in 29 patients (78 % of women who were not yet in labour). Delivery was spontaneous in 67 %, assisted vaginal (ventouse) in 14 % and by Caesarean section in 19 % of women. Perinatal deaths occurred in 30 % of women with eclampsia and 27 % of women with severe pre-eclampsia and were associated with low birth weight and prolonged time between admission and birth.

**Conclusions:**

2.4 % of women were diagnosed with severe pre-eclampsia or eclampsia. The case fatality rate and overall perinatal mortality were comparable to other reports. Better outcomes could be achieved by better treatment of hypertension and starting induction of labour as soon as possible.

## Background

Hypertensive disorders in pregnancy affect 10 % of women and are a main cause of maternal morbidity and mortality worldwide, accounting for more than 50 % of maternal deaths in sub-Saharan Africa [1, 2]. In Tanzania the maternal mortality rate is 454/100.000 of which the proportion caused by hypertensive disorders is unknown [3]. Case fatality rates (CFR) of eclampsia are reported 1–2 % in high income countries (HIC) [4, 5]. In low-income countries (LIC) CFRs vary, but are usually much higher: in two studies from Tanzanian tertiary centres CFRs of 5–8 % were reported [6, 7]. In these studies perinatal mortality was 20–39 % [6, 7].

In the recent decade attention for maternal mortality due to hypertensive disorders has been growing [8–10]. Audit of maternal morbidity and mortality cases due to hypertensive disease in pregnancy has been reported in HIC and LIC [9, 11]. Audit has been introduced at national but also at facility level, where it leads to discussion and formulation of feasible local protocols [12–15]. In a district-level hospital in rural Tanzania, maternal mortality audits are held regularly. Since 2010, the medical and labour room staff discuss all recent maternal deaths. In 2010, during such a meeting, two deaths involving women with eclampsia who died from complications following caesarean section (CS) were discussed.

Since only charts of deceased patients were available for the audit, no comparison was possible with patients with eclampsia who survived. In order to facilitate the audit process and to identify more ways to improve care of these patients, we decided to conduct a medical records study. This paper describes and analyzes the characteristics, treatment and maternal and foetal outcome of patients with severe pre-eclampsia and eclampsia treated in Ndala Hospital in 2011 and 2012, with the aim to better understand pre-eclampsia and eclampsia in this setting and to identify ways to improve care.

## Methods

This study was done at Ndala Hospital, a private Catholic hospital, situated in the Tabora region, in a rural part of Western Tanzania. It serves a catchment area of approximately 200,000 people. Annually, there are approximately 2200 deliveries in the hospital. Some women plan to give birth in the hospital, while others come after failing to give birth at home or in one of the nearby government or private health centres, up to 50 km from Ndala. There is a poor referral infrastructure, which means that many women are self-referrals from home or health centres, rarely with any written handover. Comprehensive emergency obstetric care (CEmOC) is available and conducted by three health care workers: one medical officer (medical degree) and two diploma-level assistant medical officers. There are virtually no possibilities for urgent referral to the regional hospital.

Medical records from all patients diagnosed with severe pre-eclampsia or eclampsia in Ndala Hospital between July 2011 and December 2012 were analysed. These patients were identified by the first author (RM) or one of the attending doctors. Medical records were searched immediately following discharge or death and a standard case record form was filled in by the discharging doctor and cross-checked. In case of discrepancies or missing data, the medical records were checked again. General patient characteristics, medical history, obstetrical history, possible risk factors (such as previous history, nulliparity, co-morbidities), information about the current pregnancy (gravidity, parity, gestational age (GA)), antenatal clinic (ANC) attendance and prescribed therapy before admission were recorded. Also symptoms, including blood pressure (BP), were noted on and during admission. Finally, severe complications (such as eclampsia or post-partum haemorrhage) and maternal and foetal survival were noted. The definitions that were used are given in Table 1. The hospital protocol categorises hypertension in eclamptic patients in: hypertension, severe hypertension and hypertensive crisis. If a definite diagnosis could not be made due to absent data on proteinuria, the diagnosis made by the clinician was used to determine inclusion in this study. Detection and estimation of proteinuria was done with the quantitative test using precipitation of 30 % sulfosalicylic acid [16]. Diagnosis of HELLP syndrome was not possible in the laboratory, but was suspected in case of jaundice and signs of a bleeding disorder. Magnesium sulphate (MgSO_4_) was used as anti-convulsive therapy, or could be used to prevent convulsions in patients diagnosed with severe pre-eclampsia [17]. A bolus of 4 g intravenously (IV) in 20 min was given. After that, according to the hospital protocol, MgSO_4_ was administered intravenously 4 g 4-hourly during the first year of the study. This was changed in 2012 to the same dose administered intramuscularly because of easier and safer administration. Oral antihypertensive drugs were methyldopa, hydralazine or nifedepine [18]. IV antihypertensive treatment was with hydralazine consecutive bolus injections until the BP was within predefined range (below 160/110 mmHg). Induction of labour was done with misoprostol 25 mg vaginally 8–12 hrly. No other methods of induction were used. Augmentation of (established or induced) labour with oxytocin could be considered. No cardiotocography was used.

**Table 1:** Definitions

	Definition	Inclusion criterion?^a^
Hypertension	diastolic BP ≥ 90 mmHg or systolic BP ≥ 140 mmHg	
Severe hypertension	diastolic BP ≥ 110 mmHg or systolic BP ≥ 160 mmHg	
Hypertensive crisis	diastolic BP ≥ 120 mmHg or systolic BP ≥ 180 mmHg	
Mild pre-eclampsia	Proteinuria WITH:	No
	-Hypertension WITHOUT signs or symptoms^b^	
Severe pre-eclampsia	Proteinuria WITH:	Yes
	-Hypertension AND signs, OR	
	-Severe hypertension	
Eclampsia	Hypertension	Yes
	Proteinuria	
	Convulsions	

^a^The hospital protocol categorises hypertension in hypertension, severe hypertension and hypertensive crisis. If a definite diagnosis could not be made due to absent or unreliable data on proteinuria, the diagnosis made by the clinician was used to determine inclusion in this study

^b^Signs: oedema, signs of clotting disorder, jaundice. Symptoms: headache, epigastric pain, nausea

This retrospective study was performed as part of the maternal mortality audit quality improvement activities. All data were written down by the health workers as part of routine care and after discharge collected anonymously from patient records. Because of the retrospective nature of the study, informed consent could not be obtained, but the data could not be traced back to an individual patient. Written permission and ethical clearance was obtained from the medical officer in charge as well, the district medical officer and the directorate of research and publications of the University of Dodoma (ref. UDOM/DRP/346).

Data management was done locally using Microsoft Excel®, statistical analysis was done with Epi Info®. *P*-values were calculated with *χ*^2^ (Yates corrected *P*-value), Fisher-Exact test, Mann–Whitney /Wilcoxon or *T*-test, whether appropriate.

## Results

During the study period 3398 women gave birth in the hospital. In this period 19 maternal deaths occurred. Twenty-six cases of severe pre-eclampsia and 55 cases of eclampsia were diagnosed (0.8 and 1.6 % respectively). Of all these women essential information could be retrieved. There is no method of checking whether all of the cases occurring in the study period were included. Every effort was done to include all cases as files were searched immediately after discharge. It is unlikely many cases if all have been missed.

Six women with eclampsia died (CFR eclampsia: 11 %, 32 % of all maternal deaths). Table 2 shows baseline characteristics, risk factors, signs and symptoms on admission and ANC attendance. Of all patients baseline characteristics and details about delivery could be retrieved. Women had a mean age of 22 years and most of them reported to be term.

**Table 2:** Patient characteristics

	Pre-eclampsia (*n* = 26)	Eclampsia (*n* = 55)	*P*-value
Median age (years, interquartile range)	21.5 (19–29)	20 (16–22)	0.01
Mean length of admission (days, standard deviation)	5.2 (3.3)	5.3^a^ (3.7)	0.92
Median self-reported term of pregnancy (months, interquartile range)	9 (8–9)	9 (8–9)	0.82
Nulliparity	10 (39 %)	32 (58 %)	0.16
Presenting signs and symptoms			
In labour on admission	14 (54 %)	23 (42 %)	0.44
Not in labour on admission	12 (46 %)	25 (46 %)	0.86
Delivered on admission	0	7 (13 %)	0.09
Severe hypertension or crisis	22 (85 %)	31 (56 %)	0.01
Proteinuria	18 (69 %)	37 (67 %)	0.94
Headache	7 (27 %)	28 (51 %)	0.07
Visual problems	1 (3.8 %)	8 (15 %)	0.26
Signs of clotting disorder	1 (3.8 %)	7 (13 %)	0.43
Jaundice	0	3 (5.5 %)	0.55
Oedema	16 (62 %)	23 (42 %)	0.16
Hyperreflexia	8 (31 %)	25 (45 %)	0.31
Risk factors:			
Previous pregnancy with pregnancy induced hypertension (with or without eclampsia)	2 (7.7 %)	4 (7.3 %)	1.00
Multiple gestation	4 (15 %)	5 (9.1 %)	0.46
First or new husband	19 (73 %)	38 (69 %)	0.92
None of the above risk factors	3 (12 %)	12 (22 %)	0.36
Co morbidities:			
HIV	0	1 (1.8 %)	1.00
Anaemia	1 (3.8 %)	1 (1.8 %)	0.54
Antenatal clinic visits:			
Number of women with ANC records	23 (88 %)	52 (95 %)	0.38
Of which BP or urine was checked	22 (96 %)	33 (63 %)	0.01
Median number of visits of the patients who went at least once (interquartile range)	3 (2–5)	3 (2–5)	0.42
At least 4 visits to ANC	9 (35 %)	14 (25 %)	0.56
BP checked <14 days before admission	10 (38 %)	8 (15 %)	0.03
BP diastolic <90 mmHg not more than 2 weeks ago	7 (27 %)	5 (9 %)	0.08

^a^Of the patients discharged alive (*n* = 49)

Convulsions in patients with eclampsia were classified as antepartum (44 %), intrapartum (42 %) and postpartum (15 %). Convulsions most commonly occurred (30/55, 55 %) after onset of labour or within 24 h after birth. Of the eight women (15 %) with postpartum convulsions, seven women (88 %) developed eclampsia within 24 h, one woman (1 %) had convulsions 1 week after delivery (late post-partum eclampsia). Of the 15 patients who developed eclampsia after admission, five (33 %) had normal blood pressure on admission. Proteinuria was found in 18 patients (70 %) with pre-eclampsia. In seven patients with pre-eclampsia urine was not checked and one patient tested negative for proteinuria. Of the patients with eclampsia, 37 (67 %) had proteinuria, in 15 patients (27 %) proteinuria was not checked and in three (5 %) urine was tested negative for proteinuria.

Anti-convulsive treatment was started with MgSO_4_ in all patients with eclampsia. To prevent convulsions in patients diagnosed with severe pre-eclampsia magnesium therapy was given to 22 patients (85 %) (Table 3). No serious side-effects of MgSO_4_ were reported. In three patients who received magnesium treatment for eclampsia (5 %) the magnesium was discontinued for some time. Another three patients needed a higher dose (1.5 g per hour) because of continued convulsions when on standard dose magnesium therapy.

**Table 3:** Treatment

	Pre-eclampsia (*n* = 26)	Eclampsia (*n* = 55)	*P*-value
Magnesium treatment	22 (85 %)	55 (100 %)	0.01
Of which with loading dose	18 (82 %)	50 (91 %)	0.27
Magnesium dose raised because continued convulsions	0 (0 %)	3 (5 %)	0.55
Median time of magnesium treatment (hours, interquartile range)	30 (24–38)	32 (25–50)	0.16
Antihypertensive treatment	22 (85 %)	35 (64 %)	0.07
Delivery			
Spontaneous delivery	20 (77 %)	33 (60 %)	0.21
Assisted vaginal delivery	1 (4 %)	10 (18 %)	0.09
CS	5 (19 %)	10 (18 %)	0.85
Died before delivery	0	2 (4 %)	1.00
Delivery during admission (number)	26 (100 %)	46 (84 %)	0.05
Mean time after admission (hours, standard deviation)	45 (58)	38 (58)	0.67
Women not in labour, not yet delivered	12 (46 %)	25 (45 %)	1.00
Induction misoprostol	10 (83 %)	19 (67 %)	1.00
Spontaneous in labour	2 (17 %)	5 (20 %)	1.00
Primary CS	0 (0 %)	1 (4 %)	0.70

IV antihypertensive treatment was started with hydralazine in four patients (5 %). Oral treatment with either methyldopa, hydralazine or nifedepine was given in 53 patients (65 %). In the group of 31 patients with hypertensive crisis on admission, four (13 %) received an IV antihypertensive. Twenty-one patients (68 %) used oral antihypertensives and six (19 %) did not get any antihypertensives. Of the 31 patients with hypertensive crisis, after 24 h, blood pressure was not effectively treated in eight patients (26 %), two patients (6 %) had died.

The majority of the included women (44/81, 54 %) were in labour or already had given birth. In 29 cases misoprostol was used to induce labour (36 % of all cases, 78 % of women who were not yet in labour). In 27 women induction resulted in established labour. One woman underwent CS because of failed induction and one died before getting contractions. Average time between admission and delivery in women in whom labour was induced was 68 h; 62 h in patients with eclampsia. Seven women without contractions on admission established spontaneous labour. Most women gave birth spontaneously. Assisted vaginal deliveries (ventouse) were done 11 times (14 %, see Table 3). CS was done in 15 cases (19 %), most of them (10, 67 %) because of prolonged or obstructed labour.

Of 81 women, nine (11 %) had twins resulting in 90 foetuses. Eighteen perinatal deaths (30 %) occurred in women with eclampsia and six (27 %) in women with severe pre-eclampsia (details are shown in Table 4). More than a third of the neonates of less than 2.5 kg (15/40, 38 %) died, and 6 out of 7 (86 %) of the neonates with a birth weight of less than 1.5 kg. In women with eclampsia who developed convulsions after being admitted almost all (15/16, 93 %) children survived. In women with post-partum eclampsia neonatal survival was 100 % (10/10). Of the 26 perinatal deaths (20 fresh stillbirths and 6 neonatal deaths), 11 (42 %) occurred before admission, nine (35 %) in utero during admission and six (23 %) after delivery. There was one foetal death after induction with misoprostol due to cord prolapse after spontaneous rupture of membranes. Of the six children that died between delivery and discharge of the mother, five (83 %) had a birth weight of less than 2.5 kg (average 1.71 kg). Longer duration between admission and delivery was associated with poor neonatal survival.

**Table 4:** Foetal outcomes (90 foetuses)

	Pre-eclampsia (*n* = 30)	Eclampsia (*n* = 60)	*P*-value
Alive on birth	24 (80 %)	46 (77 %)	0.93
Alive at moment of discharge	22 (73 %)	42 (70 %)	0.93
Alive at moment of discharge with mother alive	22 (73 %)	39 (65 %)	0.58
Mean birth weight (kg, standard deviation)	2.6 (0.80)	2.3 (0.69)^a^	0.12
Birth weight <2.5 kg	11 (37 %)	29 (49 %)^a^	0.37
Birth weight <1.5 kg	2 (7 %)	5 (8 %)^a^	0.91

^a^ Birth weight was available for 59 children

Of six maternal deaths, two occurred ante partum. One patient with post-partum eclampsia gave birth before admission, but died later. There was one death due to shock after complications of CS (suspected abdominal bleeding), one due to possible HELLP-syndrome and multi-organ failure and one due to hypovolaemic shock caused by post-partum haemorrhage (PPH). Three deaths were of unknown causes, possible due to embolism, intra-cerebral haemorrhage; magnesium toxicity could not always be ruled out. No autopsies were performed. In all maternal deaths diastolic blood pressure was over 110 mmHg on admission, and four had hypertensive crisis.

## Discussion

This paper describes and analyzes the characteristics, treatment and maternal and foetal outcome of patients with severe pre-eclampsia and eclampsia treated in Ndala Hospital in 2011 and 2012, with the aim to better understand pre-eclampsia and eclampsia in this setting and to identify ways to improve care. We have shown that conducting an observational study concerning pre-eclampsia within the context of audit is feasible in a rural district hospital in a LIC. The results of the study have helped hospital management to better understand what happens with patients with pre-eclampsia and eclampsia in the district and to improve the quality of care for these patients diagnosed in the hospital.

In our hospital, in contrary to hospitals in HIC, pre-eclampsia is less common than eclampsia [8]. There are a few possible explanations. Firstly, there could be selection bias. Patients with pre-eclampsia often give birth at home unnoticed and do not seek help until they get convulsions. Another explanation could be underdiagnosis of pre-eclampsia due to poor quality of BP measurements during ANC visits. The clinical picture of pre-eclampsia is heterogeneous with some women progressing fast to hypertensive crisis and convulsions, while others have an insidious rise in BP and impaired foetal growth. A third of the patients who developed eclampsia after admission had normal blood pressure measurement at the moment of admission and in five of eight women (63 %) with eclampsia who had their BP checked less than 2 weeks before admission no hypertension was recorded. The observed proportion of women with eclampsia getting convulsions after giving birth (15 %) is comparable with other LIC, but less than in HIC [4, 5, 7], probably due to many deliveries not being attended by skilled health care workers.

Since the inclusion was based on clinical diagnosis, some patients were included with the diagnosis of preeclampsia or eclampsia, while urine was not checked for proteinuria or proteinuria was absent (32 % of the cases). The method we used for diagnosing proteinuria using 30 % sulfosalicylic acid precipitation is not reliable [19], and no longer recommended [20]. Since reliable urine measurements are often not available in hospitals in LIC, our study reflects the common practice of diagnosing pre-eclampsia and eclampsia in such small rural hospitals. Although definitions exist to diagnose pre-eclampsia without proteinuria [21, 22], other diagnostic criteria are equally difficult to find in a rural setting.

Prevention of eclampsia by (early) identification and treatment of pre-eclampsia is difficult. Many women do not have obvious risk factors (only 7.4 % had a history of PIH). Signs and symptoms can be absent or present shortly: for example, 12 of 18 patients (67 %) had a normal BP within 2 weeks before admission. Increasing the number of ANC visits and BP measurements will improve detection of hypertensive disorders in pregnancy, but is a challenge in Tanzania. Small ANC clinics are often short of sphygmomanometers [23] and in rural Tanzania, though most women attend ANC regularly, many choose to give birth at home [3, 24–26]. Another problem in early detection and prevention is late booking [27]. We found a high ANC attendance of more than 90 %, but only 23 women (28 %) attended the recommended four visits [28]. In our study, in 55 % of women eclampsia occurred after onset of labour or within 24 h after birth. This means that increasing the number of hospital deliveries and ensuring qualified hospital staff can help to early recognize and treat pre-eclampsia and eclampsia.

Magnesium therapy was available during the study period and used in all patients with eclampsia and most patients with pre-eclampsia. Although severe hypertension and hypertensive crisis were common, IV antihypertensive treatment was often not installed, against the recommendations of the hospital protocol. Suboptimal BP management has been identified as a possible cause for morbidity in European countries as well [29]. The high number of women with persistent severe hypertension 24 h after admission highlights the importance of better monitoring of the patients and, if necessary, earlier and more aggressive treatment [30]. Another factor in underuse of IV antihypertensives is the unreliable stock of hydralazine in the hospital.

In 29 patients labour was induced; only one (3,4 %) needed CS because of failed induction. This is comparable to the observed success rate of Kidanto et al. [31]. The time between induction and delivery was not documented and some women started induction some time after admission, due to delayed diagnosis, delay in starting induction or because of waiting to finish steroid treatment. More emphasis needs to be put on starting induction as soon as possible after stabilisation. Our data show that almost all women will gave birth vaginally after induction. CS should be discouraged, since it poses women with severe pre-eclampsia and eclampsia under increased risk for morbidity and even mortality [32].

Use of ventouse deliveries reduced the number of CS without any complications recorded. The use of assisted vaginal deliveries is not part of standard medical practice in Tanzania [33, 34], although in tertiary centres it is used successfully [6, 7]. Ventouse deliveries are part of the Basic Emergency Obstetric Care (BEmOC) package of services, an effective strategy propagated by the WHO to address the most common direct obstetric complications [35, 36]. Performing assisted vaginal deliveries on right indications, in order to prevent (emergency) CS and its short- and long-term complications, should be a priority [37, 38].

The perinatal mortality of 30 % in eclampsia is similar to other reports. Perinatal mortality related to pre-eclampsia, 27 % in our group, is rarely studied in Africa. Since GA could not be obtained reliably, it was impossible to distinguish prematurity and small for GA. Lower GA and lower birth weight were predictors for foetal and neonatal death. Children of women with convulsions starting during admission with prompt treatment had better chances to survive.

Data from this retrospective study were collected from patient records shortly after discharge (or death), which ensures more complete and more accurate data collection than a retrospective study longer after discharge could. There are several limitations as well. Data collection was not blinded and could be subject to information bias. Some variables and outcomes are based on clinical judgement of attending doctors and not blinded for end points. Also selection bias of patients with pre-eclampsia not being identified can be suspected. These limitations are a reality in auditing pre-eclampsia and eclampsia in a small hospital.

However, very few studies are conducted in district-level facilities in LIC, while they constitute a large proportion of all health facilities and the first point of care for many patients. For that reason the authors believe that the findings from this study can be of importance for health workers working in similar conditions. Further studies should focus on evaluating the optimal management strategy for patients with pre-eclampsia and eclampsia in rural settings in LIC. Prospective studies can identify the best strategy of delivery, including timing of CS.

## Conclusion

The CFR of eclampsia was 11 % and the perinatal mortality 30 % in this rural hospital. Longer admission to delivery intervals were not associated with maternal mortality. Foetal death is associated with low birth weight and prolonged time between admission and birth. Better outcomes could be achieved by better treatment of hypertension and starting induction of labour as soon as possible (after stabilising the condition of the mother). Reliable protein measurements should be available for accurate diagnosis. Most women gave birth vaginally after induction of labour with misoprostol. This practice of first stabilising maternal condition and then opting for a vaginal birth has proven to be safe, even in those who eventually needed a CS.

## Abbreviations

ANC: Antenatal clinic
BEmOC: Basic emergency obstetric care
BP: Blood pressure
CFR: Case fatality rate
CEmOC: Comprehensive emergency obstetric care
CS: Caesarean section
GA: Gestational age
HIC: High income country
IV: Intravenous
LIC: Low income country
PE: Pulmonary embolism
PPH: Post-partum haemorrhage
UK: United Kingdom
